# Proximity-enhanced co-immobilized enzyme cascade for efficient bioconversion of nicotine to 3-succinoylpyridine

**DOI:** 10.1186/s40643-026-01066-9

**Published:** 2026-05-13

**Authors:** Yelong Wang, Jiandong Zhang, Hongjing Yang, Jinbin Wei, Kai Song, Shan Li, Laiwei Shen, Guangyu Yang, Mohamed Yassin Ali, Zhen Wang, Yong Zhang, Yuzhen Wang

**Affiliations:** 1 School of Life Sciences and Biotechnology, Inner Mongolia Agricultural University, Hohhot, 010011 Inner Mongolia China; 2 Technology Center, Gansu Tobacco Industrial Co., Ltd, Lanzhou, 730050 China; 3 State Key Laboratory of Microbial Metabolism, Joint International Research Laboratory of Metabolic and Developmental Sciences, School of Life Sciences and Biotechnology, Shanghai Jiao Tong University, Shanghai, 200240 China; 4 Department of Biochemistry, Faculty of Agriculture, Fayoum University, Fayoum, 63514 Egypt

**Keywords:** Cascade reaction, Self-assembly, Co-immobilization, NADP^+^ regeneration, 3-succinoylpyridine

## Abstract

Tobacco waste, rich in nicotine, is both an environmental burden and a potential feedstock for high-value chemicals. Here, we developed a proximity-enhanced co-immobilized multi-enzyme cascade that efficiently converts the nicotine into a pharmaceutical intermediate 3-succinoylpyridine (SP). The cascade system comprises the nicotine oxidoreductase NicA2 V321 (NicAm), pseudooxynicotine amine oxidase (Pnao), and 3-succinoylsemialdehyde-pyridine dehydrogenase (Sapd) for sequential nicotine conversion, coupled with an aldehyde–ketone reductase (AKR) module for in situ NADP^+^ regeneration. To improve nicotine-to-SP conversion and facilitate multi-enzyme recycling, a SpyCatcher/SpyTag self-assembled cofactor regeneration enzyme scaffold was adopted, in conjunction with AviTag–BirA-mediated biotinylation for cascade enzyme site-specific co-immobilization on streptavidin-coated supports. This proximity-enhanced design promoted efficient cofactor cycling and boosted the nicotine-to-SP conversion to approximately 63%, much higher than the 42.7% achieved by free enzymes. The co-immobilized system also showed improved pH and thermal stability, retaining over 60% of its initial activity after eight reuse cycles. This modular biocatalytic strategy provides a green and promising route for the valorization of nicotine-rich tobacco waste.

**Supplementary Information:**

The online version contains supplementary material available at 10.1186/s40643-026-01066-9.

## Introduction

Pyridine derivatives constitute a prominent family of heterocyclic compounds with extensive pharmacological significance, serving as versatile structural cores in numerous therapeutic agents. Among them, SP is recognized as a valuable synthetic building block, as its succinoyl‑substituted pyridine framework can be conveniently diversified into various drug‑like molecules (Naik et al. 2024; Siedentop and Rosenthal 2022). The representative applications and value of SP are illustrated in Fig. S1. Nevertheless, traditional chemical synthesis of SP and its analogs rely on petrochemical feedstocks and involves multistep redox reactions with substantial environmental drawbacks, which has driven growing interest in the development of green biotransformation approaches under mild conditions.

Meanwhile, global tobacco production and consumption generate an estimated 25 million tons of solid waste annually, which can release nicotine and other toxic substances into the surrounding ecosystem (Zafeiridou et al. 2018). Bioconversion provides a sustainable and effective strategy for converting these hazardous tobacco wastes into high-value chemicals (Gurusamy and Natarajan 2013; Yu et al. 2014). In nature, nicotine-degrading strains such as *Pseudomonas putida* metabolize nicotine via the pyrrolidine pathway, producing SP as a key intermediate (Huang et al. 2020; Liu et al. 2020; Tang et al. 2013). The detailed catalytic processes of this metabolic pathway are illustrated in Fig. S2. While biocatalytic production of SP from nicotine using genetically engineered *Pseudomonas putida* whole-cell systems has been established and currently achieves SP titers at the gram-per-liter scale (Wang et al. 2015), they typically face limitations including low cell membrane permeability, nicotine-mediated toxicity to the host strain, and the formation of undesired metabolic byproducts, all of which restrict product titers and conversion efficiency. By contrast, in vitro multi-enzyme cascade systems offer a more controllable and flexible platform (Sperl and Sieber 2018; Teshima et al. 2023). However, the practical application of multi-enzyme cascades is challenged by several drawbacks, such as the high cost of cofactor, instability of free enzymes, and the slow diffusion of intermediates between spatially separated enzymes, all of which underscore the need for spatial organization strategies (Abdallah et al. 2022; Kaushik et al. 2024; Siedentop et al. 2021; Wheeldon et al. 2016).

**Fig. 1:**
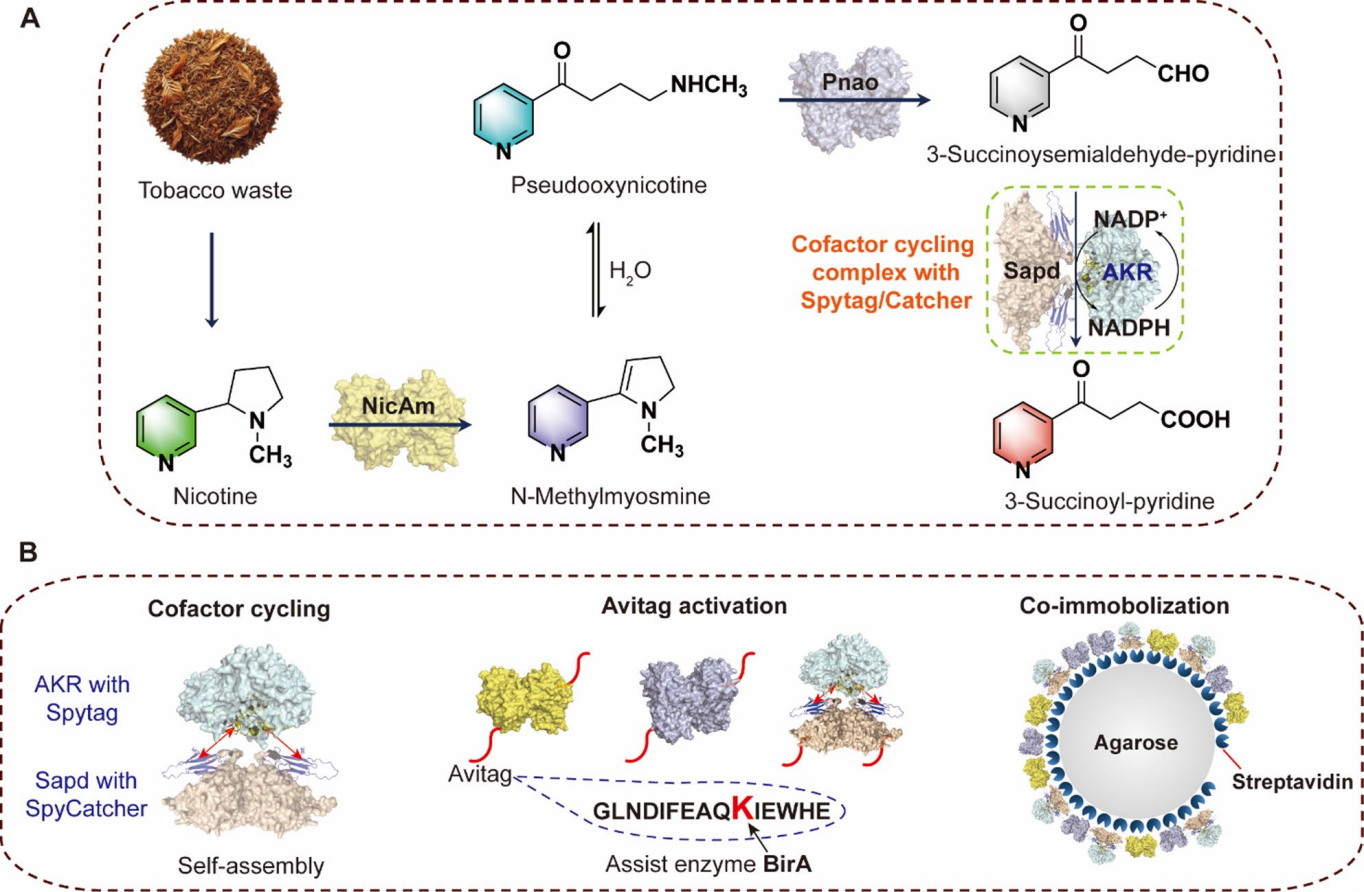
Design of an in vitro multi-enzyme cascade for the value-added transformation of nicotine from tobacco waste.** A** Nicotine obtained from tobacco waste is subjected to a multi-enzyme cascade biotransformation: nicotine is first oxidized to PN by NicAm, which is further transformed into SAP by Pnao, and then convert into SP by Sapd. The NADP^+^ cofactor recycling system is constructed via SpyCatcher/SpyTag-mediated self-assembly between Sapd and AKR, facilitating efficient in situ cofactor regeneration.** B** The Sapd–AKR self-assembled complex, together with the other cascade enzymes, was designed to be biotinylated via the AviTag/BirA system and co-immobilized onto streptavidin-coated microspheres, thereby achieving a proximity-enhanced effect and improving the reusability of the multi-enzyme cascade

To overcome these limitations, we constructed a proximity-enhanced co-immobilization of multi-enzyme cascade for the conversion of nicotine to SP, which enables the integration of pathway reconstitution, cofactor regeneration, and spatial organization engineering (Fig. 1). Our strategy exploits the SpyTag/SpyCatcher bioconjugation system and AviTag-mediated site-specific biotinylation, allowing for the precise and fast assembly of catalytic components onto solid supports (Pei et al. 2022; Peng et al. 2021; Zakeri et al. 2012). As depicted in Fig. 1, the Sapd module and AKR module were co-localized to establish an efficient in situ cofactor recycling cycle. Such spatial confinement enables efficient intermediate translocation and reduces the accumulation of unstable intermediates (Fernando et al. 2023; Yang et al. 2023).

In this work, we report the design, assembly, and functional evaluation of a proximity-enhanced co-immobilized multiple enzyme cascade for the efficient bioconversion of nicotine to SP. Our multi-enzyme cascade employs NicAm, Pnao, and Sapd as the core conversion pathway for stepwise nicotine biotransformation, complemented by an AKR-based cofactor regeneration system for sustained NADP^+^ recycling (Dulchavsky et al. 2023; Pei et al. 2020; Qiu et al. 2012). We demonstrate that spatial proximity-enhanced approach improves the cofactor recycling efficiency and catalytic performance of multiple enzyme cascade through SpyCatcher/SpyTag-mediated co-localization of Sapd and AKR, and precise, stoichiometric co-immobilization of multiple enzymes onto streptavidin–agarose microspheres. The resulting immobilized cascade exhibits enhanced bioconversion, improved operational stability, and reusability over multiple reaction cycles. Furthermore, the system was validated using a tobacco leaf crude extract as the substrate, demonstrating its potential for practical application in the valorization of real tobacco waste streams. This work thus establishes a modular, scalable, and sustainable green biocatalytic platform for conversion of tobacco nicotine into value-added pharmaceutical building blocks.

## Materials and methods

### Gene synthesis and plasmid construction

The coding sequences of *NicAm*, *Pnao*, *Sapd*, and *AKR* in previously reported studies awere chemically synthesized by Genewiz, China and codons were optimized for *Escherichia coli*. AviTag was fused to the N-terminus of each enzyme for site-specific immobilization, while SpyTag or SpyCatcher was appended to the C-terminus for covalent enzyme assembly, with each tag separated by a flexible linker (GGGGSGGGGS). All constructs were assembled by Gibson assembly and verified by DNA sequencing.

## Bacterial strains, media, and reagents

*Escherichia coli* BL21 (DE3), BL21 (DE3) pLysS, and DH5α strains were obtained from ToloBio Co. Luria-Bertani (LB) medium was used for routine cultivation and recombinant protein expression. Plasmid extraction, PCR cleanup, and homologous recombination kits were purchased from Novezan Biotech; PrimeSTAR Max polymerase from Takara Bio. PrimeSTAR Max polymerase was obtained from Takara Bio. Chemical standards of nicotine, pseudooxynicotine (PN), 3-succinoylsemialdehyde-pyridine (SAP), and 3‑succinoylpyridine (SP) were purchased from Sigma.

## Construction of recombinant expression strains

All genes encoding cascade enzymes were cloned into pET-28a by homologous recombination. Sequencing confirmed recombinant plasmids were transformed into competent *E. coli* BL21(DE3) for protein expression.

## Expression and purification of cascade enzymes

A complete list of the enzymes used is provided in Table 1. Sapd-series proteins were expressed in *E. coli* BL21(DE3) pLysS; All other proteins were expressed in *E.coli* BL21(DE3). Briefly, single colonies were inoculated into LB medium containing 50 µg/mL kanamycin and cultured at 37 °C, 220 rpm until OD_600_ reached 0.6–0.8 (~ 4 h). Protein expression and purification were carried out as previously described with minor modifications (Chen et al. 2023). Purified proteins were dialyzed overnight in Tris-HCl buffer pH 6.5 analyzed by SDS–PAGE, and stored at − 80 °C.

**Table 1:** Protein variants used in this study

Selected enzymes	Self-assembly module	AviTag module
NicAm	–	AviTag-NicAm
Pnao	–	AviTag-Pnao
Sapd	Sapd-SpyCatcher	AviTag-Sapd-SpyCatcher
AKR	AKR-SpyTag	AviTag-AKR-SpyTag

## Enzymes self-assembly and purification

To generate the Sapd–SpyCatcher/AKR–SpyTag module (with or without AviTag), clarified lysates containing each fusion protein were mixed at a 1:1 (v/v) ratio and incubated at 4 °C for 30 min. The assembled complex was purified by Ni–NTA chromatography and covalent complex formation was confirmed by SDS–PAGE.

### BirA-mediated biotinylation of AviTag proteins

AviTag-fused proteins were biotinylated using a commercial BirA enzyme kit (Beyotime Biotechnology, China). Reactions (50 µL) were assembled according to the manufacturer’s protocol, containing AviTag-fused protein, Buffer A, Buffer B, biotin, ATP, and BirA enzyme, and incubated at 30 °C for 30–60 min.

## Co-immobilization of multiple enzymes on streptavidin agarose microspheres

Streptavidin–agarose microspheres (20% slurry) were washed three times with PBS (20 mM NaH_2_PO_4_, 150 mM NaCl, pH 7.4). Biotinylated enzymes were added at amounts not exceeding the manufacturer’s binding capacity and incubated at 4 °C for 30 min with gentle stirring. Microspheres were washed three times with PBS to remove unbound proteins, and protein loading was quantified by BCA assay of flow-through fractions. Loaded microspheres were used immediately or stored at 4 °C.

## Enzyme assays and cascade reactions

Unless otherwise stated, enzyme activity assays were performed with a standard reaction condition in 50 mM Tris–HCl (pH 6.5) at 30 °C with 0.5 mM substrate, 0.2 mM NADP^+^, and 0.1–0.5 µM enzyme. Reactions were quenched after 30 min by addition of an equal volume of methanol, and supernatants were analyzed by HPLC. One unit of enzyme activity was defined as the amount converting 1 µmol substrate per minute under the above conditions. For kinetic analysis, substrate concentrations ranged from 0.01 to 5.0 mM (nicotine, PN, SAP, or acetylacetone). Enzyme cascade reactions were initiated with NicAm, Pnao, Sapd, and AKR at an equal molar ratio. Kinetic parameters were determined by nonlinear Michaelis–Menten regression. The optimal pH and temperature of multiple enzyme system were analysis over certain range using the standard reaction condition. The pH and temperature stability were examined by incubating enzymes in selected pH buffer and temperature range for 1 h. After incubation, the retained enzyme activities were measured under the optimal condition. Storage stability. Co-immobilized and free enzyme preparations were stored at 4 °C. Residual activities were examined with the enzyme standard reaction condition. All experiments were performed in biological triplicate with technical triplicate measurements. Data were analyzed and plotted using GraphPad Prism 10.

### Reactions using multiple enzyme co-immobilized microspheres

Reactions with immobilized cascades were performed in 200 µL volumes containing 100 µL enzyme-loaded streptavidin microspheres, 0.5 mM substrate, 0.2 mM NADP^+^, and 50 mM Tris–HCl (pH 6.5), incubated at 600 rpm, 30 °C. For reusability assessment, microspheres were recovered by centrifugation, washed three times with buffer, and reused for up to eight consecutive cycles. SP production was quantified by HPLC, with cycle 1 defined as 100% relative activity.

### HPLC analysis of nicotine and its degradation products

Nicotine, PN, SAP, and SP were quantified by High-performance liquid chromatography (HPLC) on an Agilent system with UV detection using an XB-Phenyl column (4.6 × 150 mm, 5 μm; Welch Materials). Mobile phase A was 0.1% triethylamine in water (pH 8.5, adjusted with phosphoric acid); mobile phase B was methanol. The gradient program was as follows: 0–3.0 min, 90% A at 0.6 mL/min; 3.0–3.5 min, 90% A at 0.6→1.0 mL/min; 3.5–7.0 min, 90→60% A at 1.0 mL/min; 7.0–14.5 min, 60→45% A at 1.0 mL/min; 14.5–15.5 min, 45→60% A at 1.0 mL/min; 15.5–18.0 min, 60% A at 1.0 mL/min; 18.0–18.5 min, 60→90% A at 1.0 mL/min; 18.5–27.5 min, 90% A at 1.0 mL/min. Column temperature was 30 °C; injection volume 10 µL; detection at 260 nm.

Sapd activity was separately assessed using a C18 column (4.6 × 150 mm, 5 μm) with an isocratic mobile phase of methanol/water (1 mM H_2_SO_4_) at 13:87 (v/v), 0.6 mL/min. All analytes were quantified using external calibration curves (0.1–0.5 mM).

### LC–MS confirmation of SP product

Product identities of PN, SAP, SP, and related intermediates were confirmed by Liquid chromatography–mass spectrometry (LC–MS) on an Agilent system equipped with an Ultimate^®^ UHPLC HILIC Amphion II column (2.1 × 100 mm, 1.8 μm). Separation was performed isocratically with 20% mobile phase A (10 mM ammonium formate in water) and 80% mobile phase B (acetonitrile) at 0.3 mL/min, 30 °C, 10 µL injection. MS detection was in ESI^+^ mode (spray voltage 3.0 kV; drying gas 400 °C, 1000 L/h; argon collision gas).

### Microscopic observation and characterization

Confocal imaging was performed on a Leica confocal microscope (20× or 40× objective) with 488 nm excitation and emission collected at ~ 525 nm. For SEM, microspheres were mounted on conductive tape, dehydrated overnight at 4 °C, sputter-coated with gold, and imaged on a Hitachi SU8600 microscope at 5 kV.

## Results and discussion

### Construction of a multi-enzyme cascade for valorization of nicotine

To develop an efficient biocatalytic platform for the value-added utilization of nicotine, we constructed an in vitro multi-enzyme cascade (Fig. 1A), inspired by the pyrrolidine pathway that involved in microbial nicotine catabolism (Zhang et al. 2022). In this cascade system, nicotine derived from tobacco waste is first oxidized to PN by NicAm. The resulting intermediate PN is then converted into SAP by Pnao, which is further oxidized by the Sapd to produce the high‑value product SP. As the redox reaction catalyzed by Sapd depends on a sustained supply of the NADP^+^ cofactor, an NADP^+^ regeneration module consisting of AKR was incorporated into this multi-enzyme biocatalytic cascade.

To achieve efficient NADP^+^ regeneration, the cofactor recycling module composed of Sapd and AKR was designed to undergo spatial colocalization using SpyCatcher/SpyTag-mediated bioconjugation. In addition, a modular co-immobilization strategy was implemented to improve the biocatalytic conversion efficiency and reusability of the multi-enzyme system. Each enzyme in the cascade was fused with an AviTag, site-specifically biotinylated by BirA, and co-immobilized onto streptavidin–agarose microspheres. This spatial confinement facilitates rapid intermediate transfer and diminishes the accumulation of reactive intermediates. Accordingly, this proximity-enhanced co-immobilization approach is intended to boost the overall catalytic efficiency and operational stability of the biocatalytic cascade.

### Expression, purification, and functional characterization of cascade enzymes

**Fig. 2:**
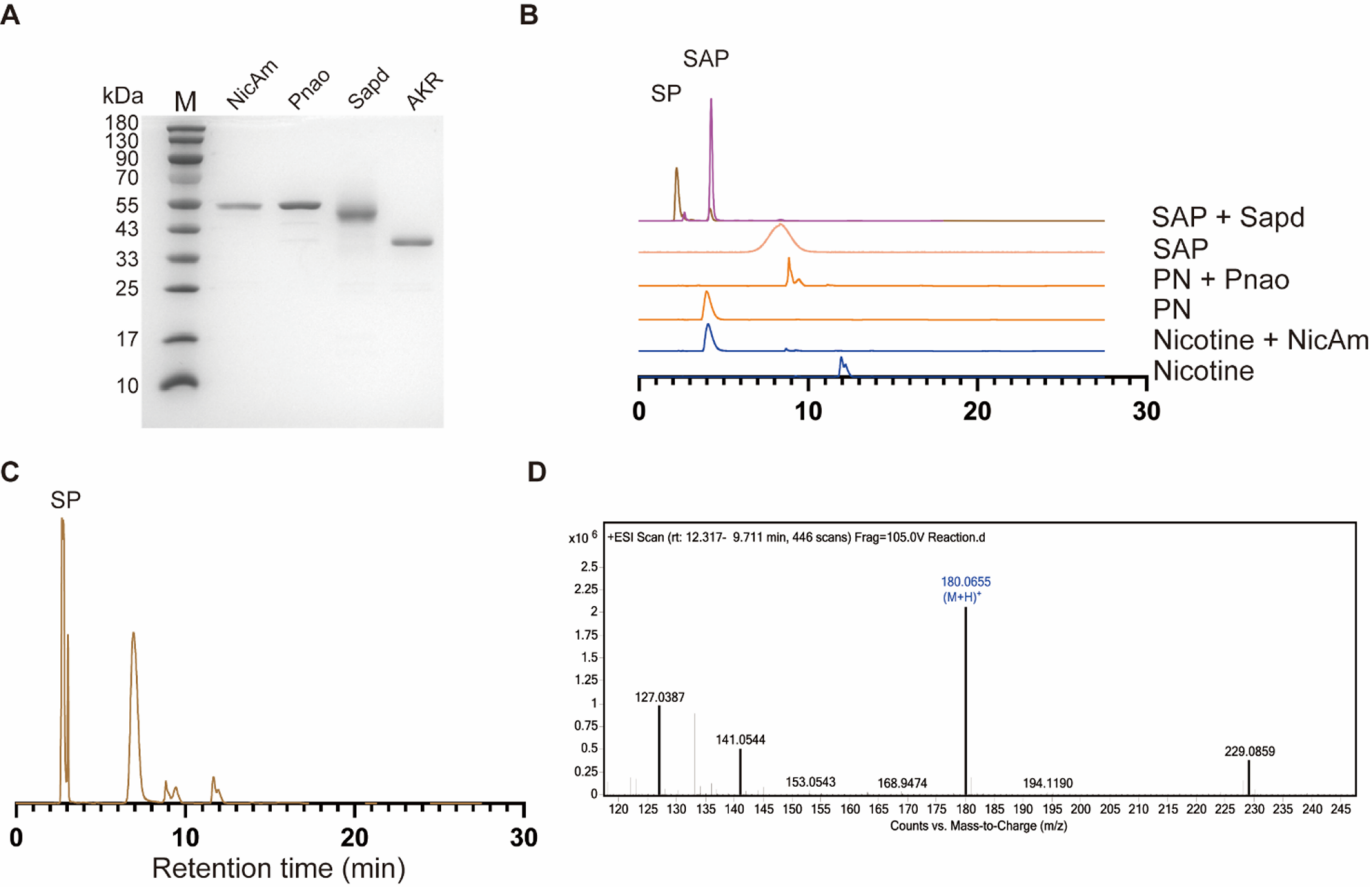
Expression and functional validation of nicotine-converting enzymes.** A** 12% SDS–PAGE analysis of NicAm, Pnao, Sapd, and AKR expression and purification.** B** HPLC chromatograms showing the catalytic activity of individual enzymes. NicAm oxidizes nicotine to PN; Pnao converts PN to SAP-aldehyde; Sapd oxidizes SAP to SP; The SAP peak in the top trace has been shifted due to the different HPLC conditions used for the analysis.** C** HPLC analysis of the reconstructed cascade shows complete nicotine consumption and SP formation, indicating successful integration of all steps.** D** LC–MS confirmation of target product SP. The observed [M + H]^+^ ion at m/z 180.0655 matches the theoretical molecular weight of SP

To establish the designed cascade catalytic system, NicAm, Pnao, Sapd, and AKR were recombinantly expressed and purified. SDS‑PAGE analysis verified the preparation of all four enzymes, with their apparent molecular weights consistent with theoretical predictions (Fig. 2A). The catalytic activity of each enzyme was initially evaluated by HPLC (Fig. 2B). NicAm catalyzed the oxidation of nicotine to PN, as reflected by the significant reduction of the nicotine peak and the emergence of a clear PN peak. PN was subsequently transformed into SAP by Pnao and further oxidized to SP by Sapd. The identity of the product and intermediates was confirmed by comparison with reference standards under the corresponding HPLC analysis conditions.

Next, we reconstructed the complete cascade in vitro and assessed the overall conversion performance. As shown in Fig. 2C, the assembled cascade led to efficient substrate consumption with concomitant formation of SP as the dominant product peak, demonstrating successful integration of all catalytic steps. To further characterize the cascade’s flux, we monitored the accumulation of key intermediates over time (Fig. S4). The HPLC results revealed that PN and SAP dynamically accumulate and are subsequently consumed, validating the sequential nature of the multi-enzyme conversion. The identity of SP was verified by LC–MS analysis, where the detected [M + H]^+^ ion at m/z 180.0655 matched the theoretical molecular ion of SP (Fig. 2D), providing definitive confirmation of the target product. We also examined the initial enzyme profile for cofactor regeneration, and observed that Sapd exhibits relatively lower catalytic efficiency compared with AKR (Fig. S3). To optimize the enzymatic cascade, we explored the multi-enzyme reaction by adjusting the enzyme ratios. The results revealed a relatively optimal ratio of 1:3:6:3 (NicAm: Pnao: Sapd: AKR) for the production of 3-succinoylpyridine (Fig. S7).

### Construction of an efficient cofactor regeneration module via spytag/spycatcher-mediated self-assembly

**Fig. 3:**
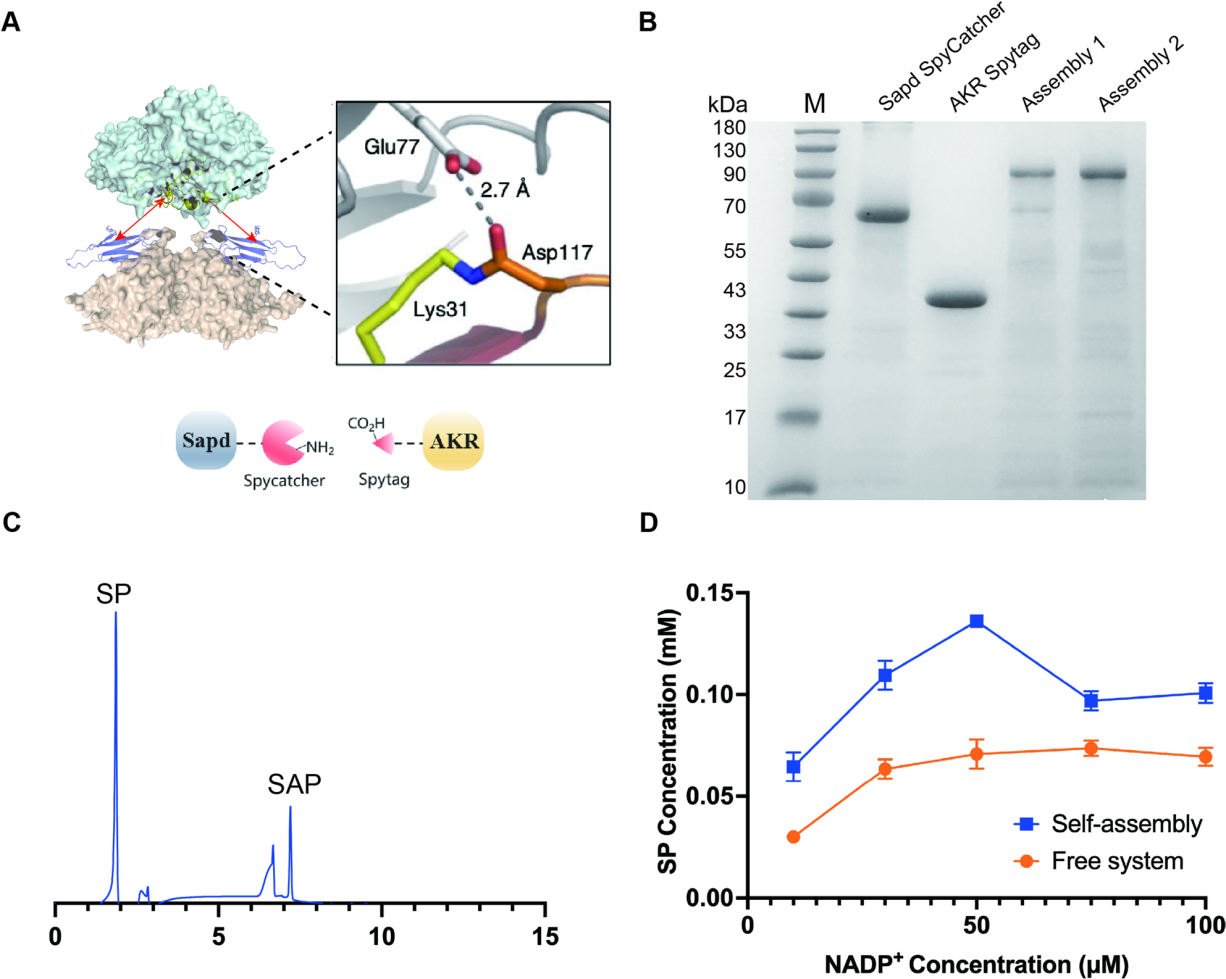
Functional verification of self-assembled Sapd-AKR cofactor-regeneration module. **A** Schematic of the self‑assembly design for Sapd and AKR: SpyTag/SpyCatcher‑mediated rapid and irreversible assembly of Sapd and AKR to promote NADP^+^/NADPH turnover.** B** 12% SDS–PAGE analysis of the covalently self-assembled enzymes. Lane M, molecular weight marker; Lane 1, the purified Sapd-SpyCatcher; Lane 2, the purified AKR-SpyTag; Lanes 3–4, self-assembled Sapd-AKR enzyme complex.** C** HPLC analysis examining the function of the self‑assembled NADP^+^ regeneration module during the conversion of SAP to SP.** D** Comparison of the effect of NADP^+^ concentration (10–100 µM) on SP formation by the free enzymes and the self‑assembled Sapd-AKR. The self‑assembled enzyme complex produced higher levels of SP across all tested NADP^+^ concentrations, indicating proximity channeling driven cofactor recycling

Previous studies have demonstrated that proximity or scaffold-mediated assembly can improve metabolic pathway efficiency by elevating the local effective concentrations of cooperative enzymes and minimizing intermediate diffusion distances (Jiang et al. 2026; Lu et al. 2026; Wang et al. 2023). The SpyTag/SpyCatcher system enables spontaneous formation of stable isopeptide bonds, facilitating rapid and irreversible enzyme assembly for improved cascade catalysis (Ali et al. 2021; Yin et al. 2018). To ensure efficient cofactor supply within the enzymatic cascade, we designed the cofactor‑recycling enzymes Sapd and AKR to be expressed as fusions with SpyTag and SpyCatcher, respectively. These fusion enzymes facilitate the self‑assembly of the enzyme complex and locates Sapd in close vicinity to the redox center of AKR (Fig. 3A). Molecular docking was first conducted using AlphaFold to predict the distance between the redox centers of Sapd and AKR in the assembled complex. By inserting a linker peptide (GGGGSGGGGS) between SpyCatcher/SpyTag and the respective enzymes, we constructed a multiple enzyme assembly complex with a predicted inter-center distance of ~ 15.8 Å (Fig. S6), which supports the structural feasibility of efficient intermediate channeling. The spatial proximity channeling is expected to promote localized NADP^+^/NADPH cycling, thereby enhancing the effective driving force for the sequential oxidation reaction.

The fused SpyCatcher/SpyTag enzymes were expressed and purified individually and then mixed to initiate self-assembly. SDS-PAGE analysis revealed a high-molecular-weight band above 170 kDa (Fig. 3B), consistent with the theoretically calculated molecular weight (165.5 kDa) of the covalently linked Sapd–AKR complex, confirming successful formation of the self-assembled enzyme complex. We next examined whether the assembled module retained its catalytic activity. HPLC analysis showed that the self-assembled Sapd–AKR complex efficiently converted SAP to SP (Fig. 3C), demonstrating that the Spy-mediated coupling enzyme complex exhibits catalytic function comparable to that of unassembled components.

To further evaluate whether the self‑assembled multi-enzymes module exerts a spatial‑proximity channeling effect that improves cofactor cycling, we compared the free enzyme mixture with the self‑assembled Sapd-AKR complex across a range of NADP^+^ concentrations (10–100 µM). As depicted in Fig. 3D, the self-assembled Sapd-AKR module yielded higher levels of SP relative to the free enzymes over the examined range of NADP^+^ concentrations. This observation demonstrates that proximity effect of assembly improves the efficiency of NADP^+^ utilization and reinforces the catalytic flux in reaction. In addition, the covalent linkage minimizes complex dissociation during handling, providing a practical advantage for subsequent multiple enzyme co-immobilization.

### Co-immobilization of the enzymatic cascade enhances both its biocatalytic efficiency and stability

Enzyme immobilization has been broadly employed to enhance operational stability and facilitate the reuse of biocatalysts (Sheldon and van Pelt 2013). We therefore explored an AviTag–biotin/streptavidin coupling strategy for co-immobilization of the nicotine-to-SP cascade enzymes on agarose microspheres. AviTag is a 15-amino-acid peptide that can be site-specifically biotinylated at a defined lysine residue by BirA in the presence of ATP and biotin, providing a reproducible conjugation handle while minimizing perturbation to the fusion proteins (Fairhead and Howarth 2015). The specific, high‑affinity interaction between biotin and streptavidin provides extraordinarily robust binding, enabling rapid and stable enzyme immobilization under various conditions.

**Fig. 4:**
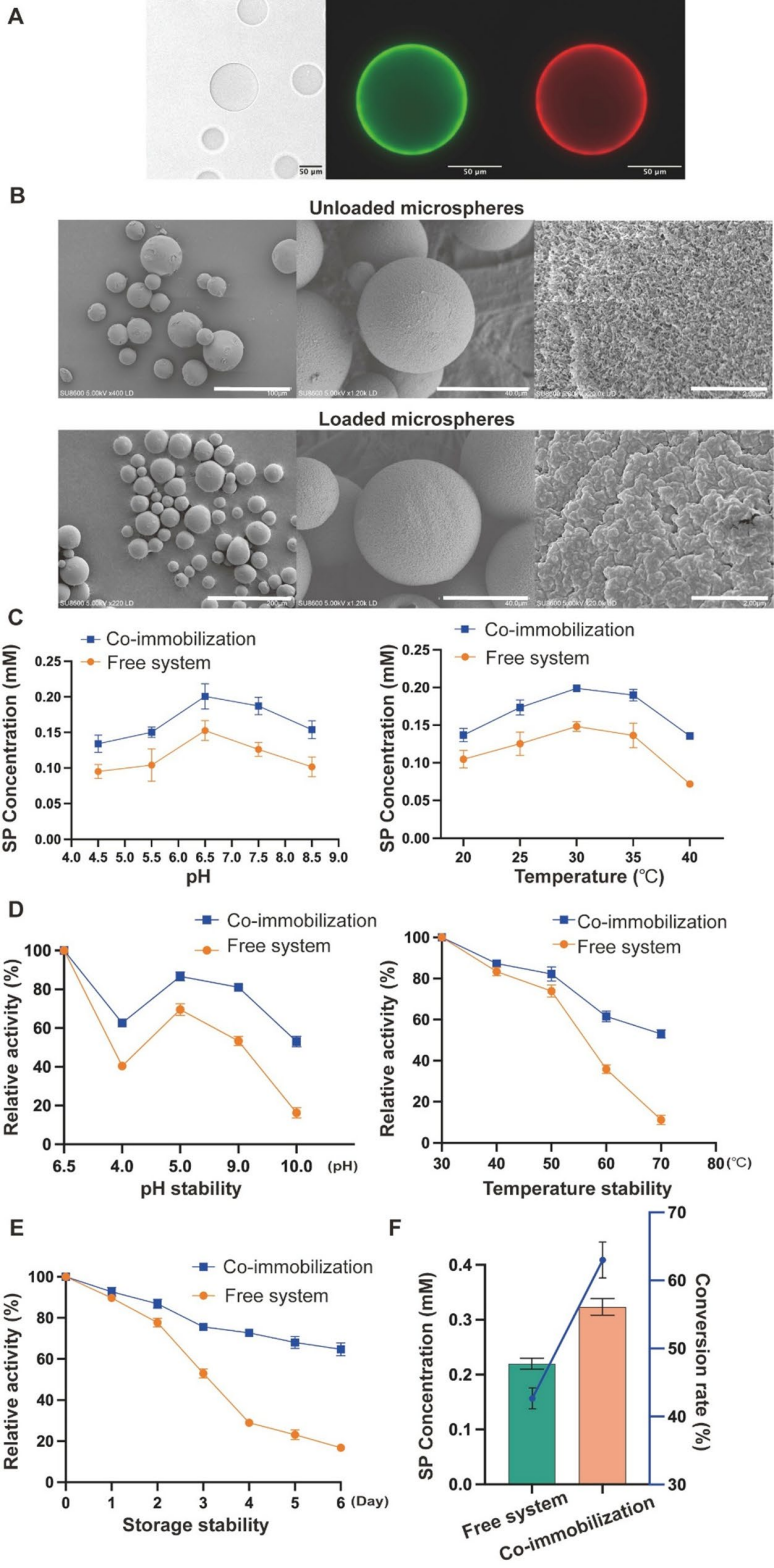
Microscopic characterization and catalytic performance of the co-immobilized multi-enzyme cascade system. **A** Confocal laser scanning microscopic analysis of co-immobilized microsphere protein loading with mNeonGreen and mCherry as fluorescent reporters. The uniform presence of distinct green and red fluorescence throughout the microsphere surface verifies the successful co‑immobilization of the multiple proteins.** B** SEM analysis was performed on both unloaded microspheres and those loaded with multiple enzymes. The unloaded microspheres exhibited smooth surfaces, while the enzyme‑immobilized microspheres showed a continuous granular layer, confirming the formation of an enzyme coating. C Optimal pH and temperature analysis of the co-immobilized and free enzyme systems.** D** pH and temperature stability of the co-immobilized and free enzymes.** E**. Storage stability comparison between the co-immobilized enzymes and free enzymes (**F**) Conversion efficiency from nicotine to 3‑succinoylpyridine (SP). The left and right Y‑axes denote the final titer of SP (mM) and the substrate conversion ratio (%), respectively

To confirm that selected microspheres can support efficient surface immobilization via this chemistry, we initially immobilized fluorescent proteins with distinct emission wavelengths and visualized the microspheres using confocal microscopy. Microspheres lacking protein conjugation showed only negligible fluorescence (Fig. 4A), verifying low background autofluorescence. In contrast, microspheres subjected to AviTag–biotin/streptavidin coupling exhibited intense, surface-restricted fluorescence signals in both detection channels (488 nm and 525 nm wavelength), indicating homogeneous protein distribution across the microsphere surface (Fig. 4A).

The multi-enzyme cascade was immobilized onto functionalized microspheres through a site-specific biotin-streptavidin interaction. Purified AviTag-NicAm, AviTag-Pnao, and the pre-assembled AviTag-Sapd SpyCatcher-AKR SpyTag complex were firstly biotinylated using BirA biotin ligase. These components were mixed and optimized and then incubated with microspheres at 4 °C to complete the co-immobilization. Quantitative analysis of the carrier’s performance demonstrated that its protein loading capacity reached 0.3611 mg/g. (Table S3).

The surface morphology of the microspheres after enzyme loading was further investigated via scanning electron microscopy (SEM). Relative to the unloaded support, microspheres loaded with multiple enzymes displayed a continuous and comparatively rough granular surface microstructure, which implied that an outer enzyme layer had been successfully coated onto the microspheres (Fig. 4B). The microscopic observation offers supplementary evidence supporting the successful co-immobilization of proteins onto the microspheres.

To assess the catalytic performance of the enzyme following immobilization, the kinetic parameters of both free and immobilized enzyme preparations were determined. The results indicated that the apparent Michaelis constant *K*_m_ of immobilized Spad‑SpyCatcher could not be determined, which may be attributed to the low chemical stability of SAP. For all other immobilized enzymes, their apparent *K*_m_ values showed a slight increase (Table S1), while the overall catalytic efficiencies (*k*_cat_ /*K*_m_) remained comparable to those of the corresponding free enzymes. These findings imply that co-immobilization of multiple enzymes through the AviTag-BirA system is capable of preserving the active conformation of the enzymes.

To distinguish the effect of directed spatial organization from immobilization effects, a random co-immobilization control was examined (Fig. S5). The specifically assembled SpyTag/SpyCatcher system outperformed the randomly co-immobilized enzymes, verifying that enhanced spatial proximity improved cascade flux. The co-immobilized multi-enzyme cascade system displayed an optimal pH of 6.5 and an optimal temperature of 30 °C, which were comparable to those of the free enzyme system (Fig. 4C). To further assess operational robustness, the pH temperature stability of both systems was evaluated. The co-immobilized cascade demonstrated markedly enhanced tolerance to a broad range of pH (6.5–10.0) and elevated temperatures (30–70 °C) compared to free enzymes (Fig. 4D). Following incubation at 70 °C, the co‑immobilized system retained more than 50% of its relative activity, while the free enzymes preserved only around 10% activity. In addition, the co‑immobilized system displayed better storage stability: it maintained roughly 65% of its initial activity after 6 days at 4 °C, whereas the activity of the free system dropped to 20%.

**Fig. 5:**
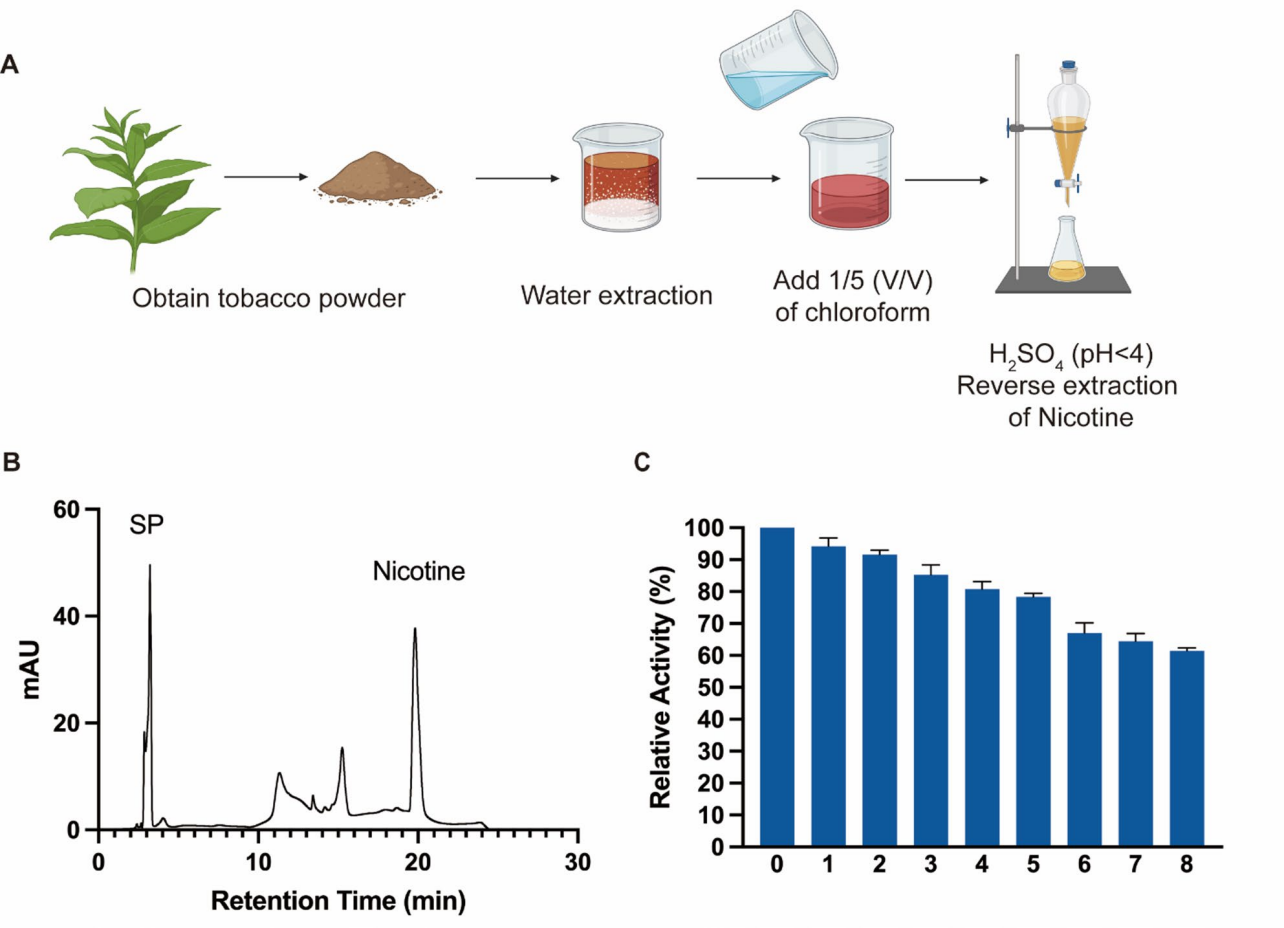
Preparation of tobacco leaf–derived nicotine and reusability of the immobilized multi-enzyme cascade. **A** Schematic of nicotine extraction from tobacco leaves via water extraction, chloroform partitioning, and H_2_SO_4_ back-extraction (pH < 4).** B** HPLC analysis of tobacco nicotine biotransformation reaction catalyzed by the co-immobilized enzymes.** C** Operational stability of the immobilized system over consecutive cycles. Relative activity was determined based on SP formation, with the first cycle defined as 100%. The system retained > 60% activity after eight cycles. Data represent mean ± SD (*n* = 3)

Finally, we quantified both nicotine conversion and target SP formation. The data revealed that the co‑immobilized enzyme cascade increased both the nicotine conversion and the final SP titer compared with the free‑enzyme system (Fig. 5E). This improved substrate conversion and SP product formation can be attributed to the improved catalytic efficiency and robustness conferred by the proximity‑enhanced co‑immobilization of enzyme cascade.

### Application of the proximity‑enhanced co-immobilized enzyme cascade to tobacco-derived nicotine and evaluation of reusability

To demonstrate practical applicability, nicotine was extracted from tobacco leaves and used as a substrate for the immobilized cascade. Dried tobacco leaves were ground, subjected to water extraction, and processed by to obtain an aqueous nicotine fraction (Fig. 5A). HPLC analysis of two independent tobacco samples revealed nicotine contents of 3.318 and 3.351% (w/w), with an average nicotine content of 3.380% (w/w) (Table S4), establishing a realistic substrate load for downstream biotransformation.

Following treatment of tobacco leaf extract with the co‑immobilized enzyme system, the resulting SP product was analyzed via HPLC. As illustrated in Fig. 5B, distinct peaks corresponding to the product SP formation were observed, confirming the nicotine biotransformation into SP by co-immobilization system. Then we assessed the operational stability of the co-immobilized multiple enzyme cascade system over multiple consecutive reaction cycles using tobacco leaf nicotine. As presented in Fig. 5C, the catalyst maintained relative high activity across the eight cycles tested, retaining more than 60% of its initial activity. To investigate whether this activity loss was attributable to enzyme leaching, a leaching assay was performed after eight reuse cycles. More than 87% of the immobilized protein was retained on the microspheres after the eighth cycle (Table S2), suggesting that protein leaching was not the primary cause of the observed activity decay.

## Conclusion

In this study, we developed a robust, proximity-guided multi-enzyme cascade for the efficient bioconversion of nicotine into the high-value intermediate SP. By using NicAm, Pnao, and Sapd, and integrating an AKR-based cofactor regeneration module, we achieved quantitative nicotine consumption and selective SP formation. The spatial co-localization of Sapd and AKR via SpyCatcher/SpyTag bioconjugation significantly enhanced local NADP^+^/NADPH recycling efficiency. Combined with AviTag–BirA-mediated site-specific biotinylation and streptavidin-agarose immobilization, this system exhibited superior pH and temperature stability, delivering an SP titer of ~ 0.35 mM from 0.5 mM substrate (~ 70% conversion) with an approximately 1.6-fold increase in output compared to the non-immobilized counterpart. The co-immobilized biocatalysts demonstrated excellent operational stability when processing industrial tobacco extracts, retaining over 60% of its initial activity after eight consecutive cycles. Given the significant nicotine content in tobacco waste, these results offer a modular biocatalytic strategy for transforming tobacco nicotine into valuable pharmaceutical precursors.

## Supplementary Information

Below is the link to the electronic supplementary material.

Supplementary Material 1.

## Data Availability

All data analyzed during this study are included in this article. The data is available from the corresponding author upon reasonable request.
