# Supplementary material for: Proximity-enhanced co-immobilized enzyme cascade for efficient bioconversion of nicotine to 3-succinoylpyridine
Source: Bioresour Bioprocess. 2026 May 13;13(1):70. doi: 10.1186/s40643-026-01066-9 (PMC13172129; doi:10.1186/s40643-026-01066-9)
Supplement: Supplementary file 1 — Supplementary Material 1. [file 40643_2026_1066_MOESM1_ESM.docx]

**Supporting information**

**Proximity-enhanced Co-Immobilized Enzyme Cascade for Efficient Bioconversion of Nicotine to 3-Succinoylpyridine**

Yelong Wang^1,3^, Jiandong Zhang^2^, Hongjing Yang^2^, Jinbin Wei^2^, Kai Song^2^, Shan Li^2^, Laiwei Shen^3^, Guangyu Yang^3^, Mohamed Yassin Ali^3,4^, Zhen Wang^2*^, Yong Zhang^3*^, and Yuzhen Wang^1*^

^1^ School of Life Sciences and Biotechnology, Inner Mongolia Agricultural University, Hohhot, Inner Mongolia, 010011, China

^2^ Technology Center, Gansu Tobacco Industrial Co., Ltd., Lanzhou, 730050, China

^3^ State Key Laboratory of Microbial Metabolism, Joint International Research Laboratory of Metabolic and Developmental Sciences, School of Life Sciences and Biotechnology, Shanghai Jiao Tong University, Shanghai 200240, China.

^4^ Department of Biochemistry, Faculty of Agriculture, Fayoum University, Fayoum 63514, Egypt

*Corresponding author(s). Email (s): wangyuzhen@imau.edu.cn; yzhang2011@sjtu.edu.cn; 361514540@qq.com;

**

Figure S1.** **3-Succinoyl-pyridine (SP) as a platform N-heterocycle for drug-oriented synthesis.**
SP (center) is highlighted as a versatile pyridine synthon. Dashed arrows radiating from SP indicate representative valorization routes to drug classes: Vitamin B6 (pyridoxine); hypotensive agents of the ω-heteroaroyl-(propionyl)-L-proline family (general R₁–R₄ substituent scope); analgesics such as propiram; the nicotinoyl–γ-Aminobutyric acid prodrug picamilon with hydrolytic release of γ-Aminobutyric acid (GABA) (blue dashed arrow); anti-inflammatory drugs clonixin and nicoboxil; 6-hydroxy-3-succinoyl-pyridine (HSP) as a derivatization handle leading to an epibatidine-like analgesic scaffold (blue dashed arrow); and analeptics including nicethamide (and its quaternized analog) and camphothamide. Colored rings mark the N-heteroaromatic cores retained across transformations. Abbreviations: Me, methyl; Et, ethyl; NEt₂, diethylamide; OEt, ethoxy; Bu, butyl; HSP, 6-hydroxy-3-succinoyl-pyridine.


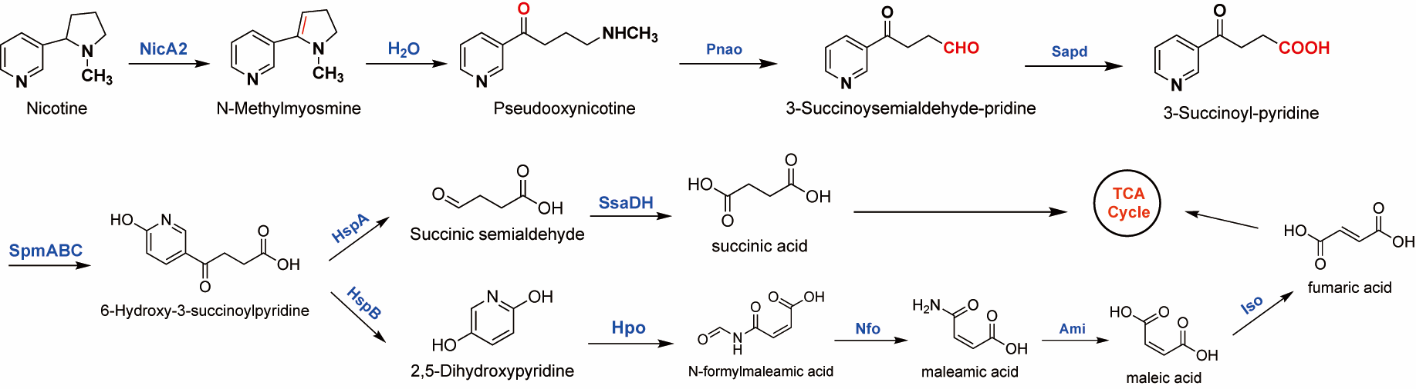
**Figure S2. A simplified scheme of pyrrolidine pathway in the natural pathway of microbial nicotine degradation.**

**
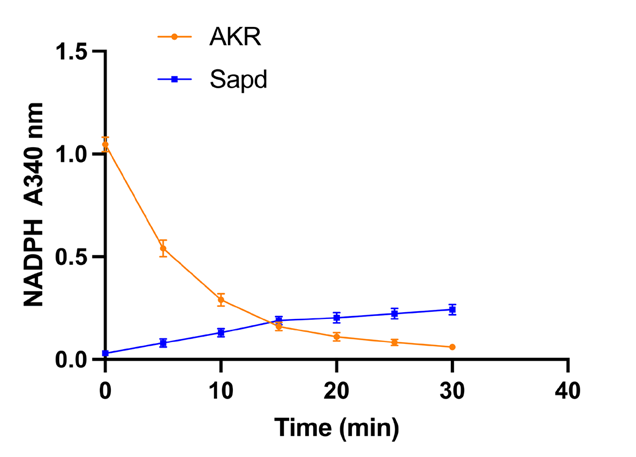
**

**Figure S3. Time-course comparison of catalytic rates for AKR and Sapd monitored by NAD(P)H absorbance.**The catalytic activities of AKR and Sapd were evaluated by continuously tracking the change in absorbance at 340 nm, corresponding to the oxidation/reduction of NADPH during the reaction. Measurements were collected at the indicated time points (0–30 min) under identical conditions (buffer: Tris-HCl pH 6.5, temperature: 25 °C). Data are presented as mean ± SD (n = 3 independent replicates).

**
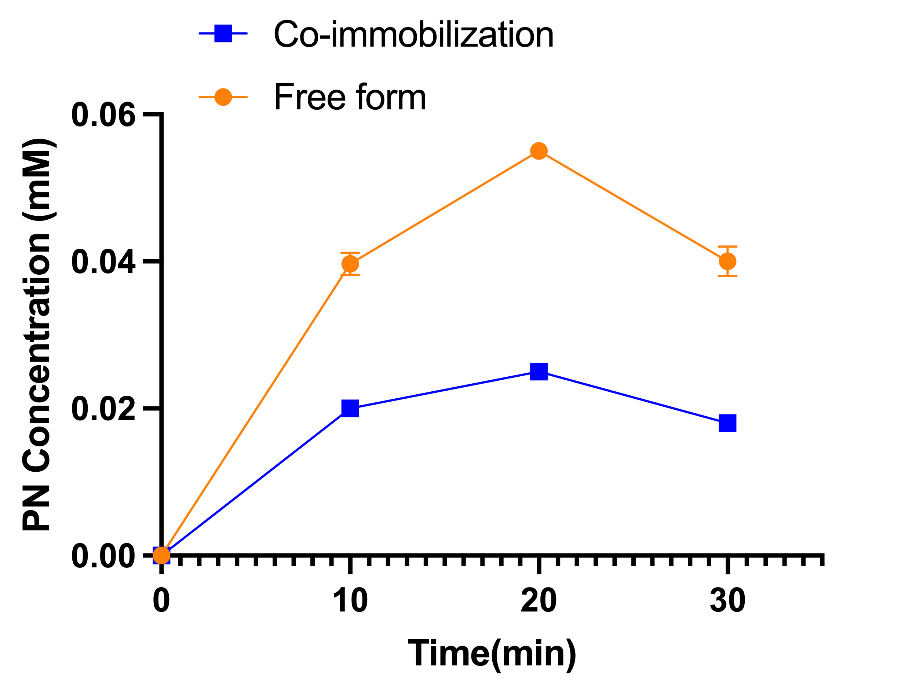
A**

**
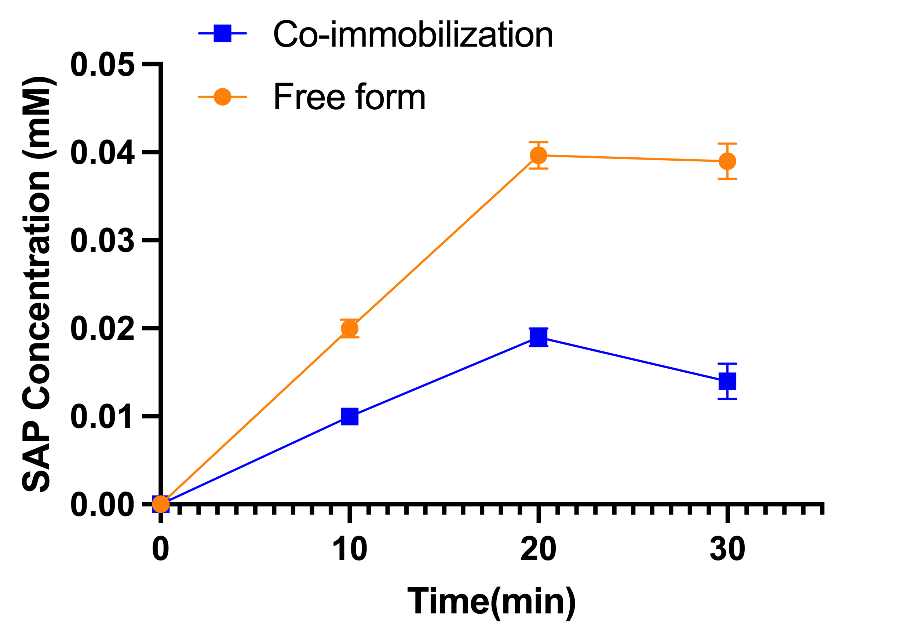
B**

**Figure S4. Time-course accumulation profiles of key intermediates in the nicotine-to-SP cascade.**
**(A)** Concentration of **pseudooxynicotine (PN)** over time during the cascade reaction for the **free-enzyme** and **immobilized** systems.
**(B)** Concentration of **3-succinylsemialdehyde-pyridine (SAP)** over time during the cascade reaction for the **free-enzyme** and **immobilized** systems.
Reactions were initiated with **0.5 mM nicotine** and samples were collected at the indicated time points. Intermediate concentrations were quantified by HPLC using authentic standards (see Methods). Data are presented as **mean ± SD (n = 3)**.

**
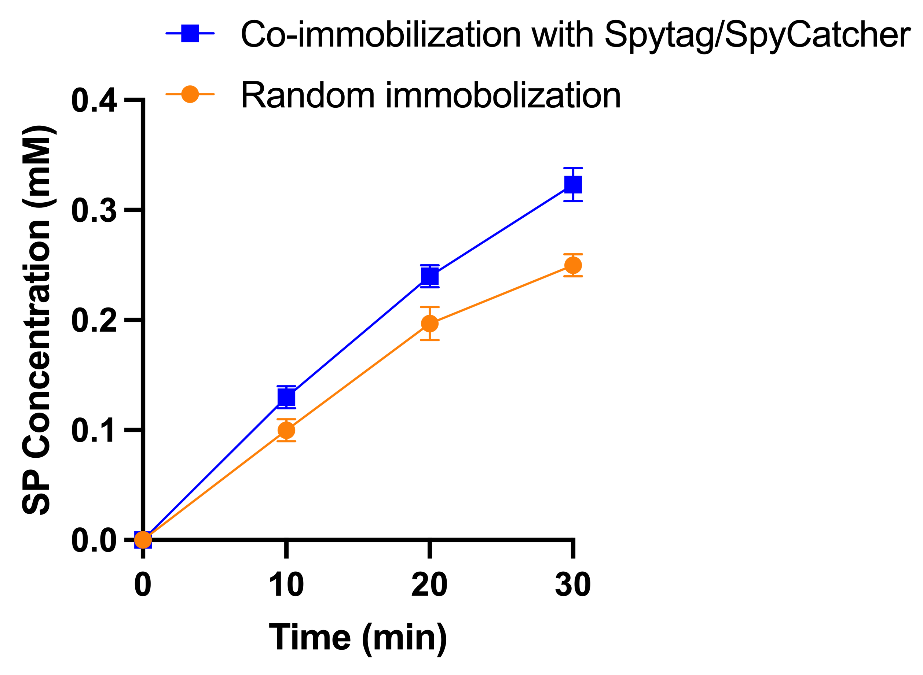
**

**Figure S5. Control experiment using random co-immobilization to isolate the contribution of spatial guidance.**
To distinguish the performance improvement derived from *spatially guided assembly* (SpyTag/SpyCatcher-mediated proximity) from the general benefits of immobilization (e.g., increased local concentration and enhanced stability), a **random co-immobilization** control was constructed by co-anchoring Sapd and AKR onto the same support **without SpyTag/SpyCatcher assembly** (i.e., no defined spatial organization). The catalytic performance of the proximity-guided co-immobilized system and the random co-immobilized control was compared under identical reaction conditions (substrate: [Nicotine], buffer: [Tris-HCl], pH [6.5], temperature: [25] °C). Data are shown as mean ± SD (n = 3 independent replicates).

**
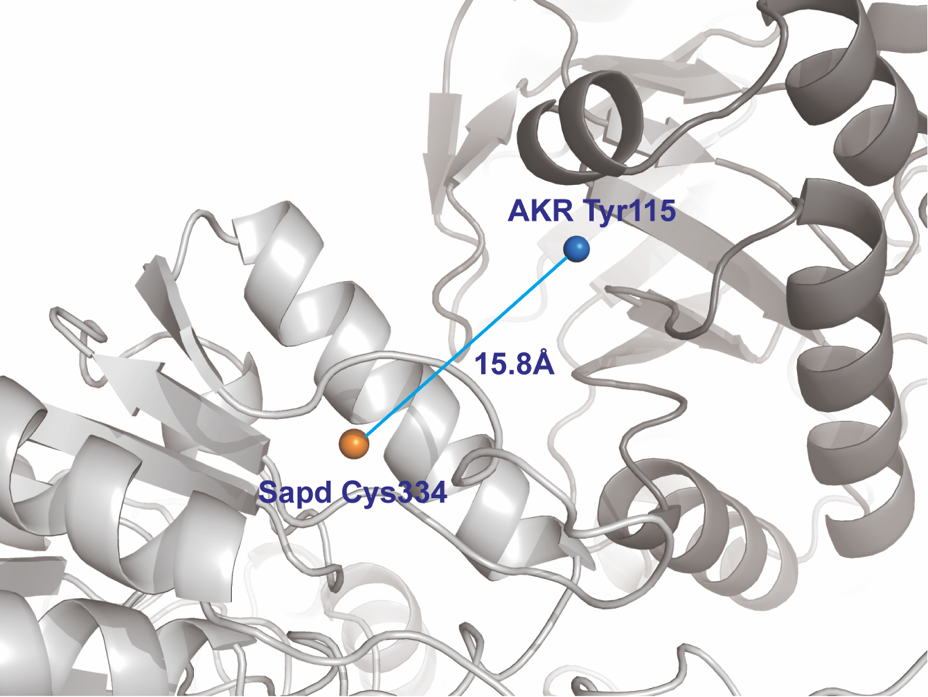
**

**Figure S6. Protein docking-based estimation of the spatial distance between the Sapd and AKR redox centers.**
A representative protein–protein docking model of Sapd and AKR (workflow described in Methods) was used to estimate the spatial proximity of the two enzymes in the assembled complex. The putative redox centers were defined by the key catalytic residues **Sapd Cys334 (SG atom)** and **AKR Tyr115 (OH atom)**, which are highlighted as spheres. The inter-center distance was measured in PyMOL as the shortest distance between the selected atoms, yielding an estimated separation of **15.8 Å** (red dashed line). This docking-based measurement supports the feasibility of proximity-enabled cofactor transfer/channeling between Sapd and AKR in the proximity-guided module.

**
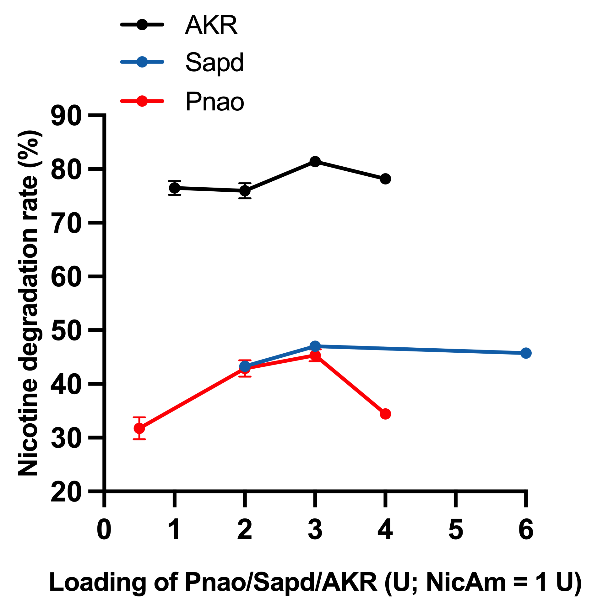
A B**

**
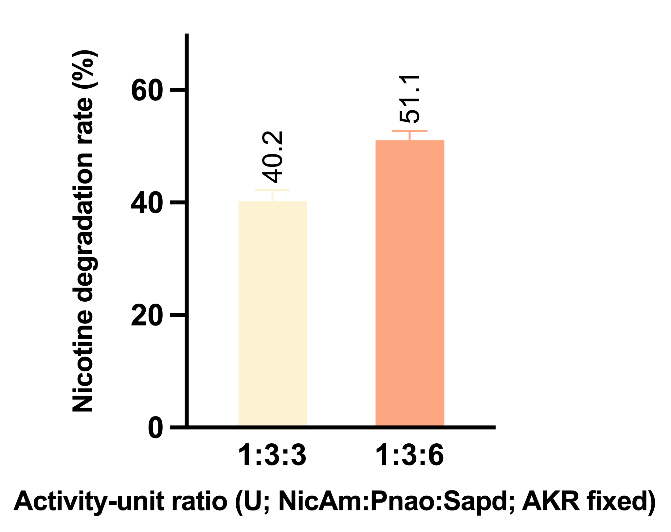
**

**Figure S7. Preliminary optimization of enzyme dosing in the multi-enzyme cascade based on activity units (U).**(A) Single-enzyme titration. The loading of one enzyme (Pnao, Sapd, or AKR) was varied (in U) while keeping the other enzymes constant (NicAm was fixed at 1 U), and the nicotine degradation rate was quantified.
(B) Co-optimization of the upstream three enzymes. The U-based ratio of NicAm : Pnao : Sapd was screened while AKR was fixed at 3 U, and the ratio 1:3:6 (thus giving an overall cascade ratio of 1:3:6:3 for NicAm : Pnao : Sapd : AKR) showed the best performance among the tested conditions. AKR was not systematically optimized in panel B because it primarily serves as a cofactor-regeneration/supporting enzyme; therefore, only a rough, preliminary setting (3 U) was used during ratio screening of NicAm, Pnao, and Sapd.

| Enzyme | *K*_m_ ^a^ | *k*_cat_ (s^-1^)^a^ | *k*_cat_/*K*_m_ (M^-1^s^-1^) |
| --- | --- | --- | --- |
| NicAm | 1.11 ± 0.02 μM | 0.68 ± 0.01 | 6.12 × 10^5^ |
| Pnao | 0.24 ± 0.03 mM | 149 ± 17 | 6.21 × 10^5^ |
| Sapd | 2.12 ± 0.09 μM | 0.45 ± 0.02 | 2.12 × 10^5^ |
| AKR | 6.13 ± 0.21 mM | (4.18 ± 0.12) × 10^3^ | 6.82 × 10^5^ |
| Sapd-SpyCatcher | 2.11 ± 0.03 μM | 0.43 ± 0.01 | 2.04 × 10^5^ |
| AKR-Spytag | 6.08 ± 0.07 mM | (4.17 ± 0.09) × 10^3^ | 6.86 × 10^5^ |
| Avitag-NicAm | 1.10 ± 0.01 μM | 0.66 ± 0.01 | 6.00 × 10^5^ |
| Avitag-Pnao | 0.24 ± 0.01 mM | 147 ± 14 | 6.13 × 10^5^ |
| Avitag-Sapd-SpyCatcher | 2.11 ± 0.05 μM | 0.44 ± 0.01 | 2.09 × 10^5^ |
| Avitag-AKR-Spytag | 6.11 ± 0.13 mM | (4.18 ± 0.04) × 10^3^ | 6.84 × 10^5^ |
| Avitag-NicAm* | 1.25 ± 0.08 μM | 0.57 ± 0.03 | 4.56 × 10^5^ |
| Avitag-Pnao* | 0.32 ± 0.03 mM | 135 ± 11 | 4.22 × 10^5^ |
| Avitag-Sapd-SpyCatcher* | - | 0.41 ± 0.02 | - |
| Avitag-AKR-Spytag* | 6.25 ± 0.24 mM | (4.03 ± 0.06) × 10^3^ | 6.45 × 10^5^ |

^a^*Apparent kinetic parameters (K_m_,app, k_cat_,app,) determined from Michaelis–Menten fitting under the specified assay conditions.*

*** *Kinetic parameters of the immobilized enzymes.*

*-* *Data determinations failed or could not be calculated.*

**Table S1. Kinetic parameters of the enzymes used in the nicotine-to-SP cascade.**The Michaelis–Menten parameters (*K*_m_, *k*_cat_) and catalytic efficiency (*k*_cat_/*K*_m_) were determined for each enzyme in both free and immobilized form. The substrates used for kinetic assays were: nicotine for NicAm, pseudooxynicotine (PN) for Pnao, 3-succinylsemialdehyde-pyridine (SAP) for Sapd, and acetylacetone for AKR. Values are reported as mean ± SD from three independent measurements (n = 3). kcat is presented in s⁻¹ and *k*_cat_/*K*_m_ in M⁻¹·s⁻¹.

| Cycle | Cumulative leached (mg/g) | Remaining bound (mg/g) | Remaining bound (%) |
| --- | --- | --- | --- |
| 0 | 0.0000 | 0.3600 | 100.00% |
| 8 | 0.0440 | 0.3160 | 87.78% |

**Table S2. Leaching evaluation of immobilized enzymes during repeated reuse cycles.**A leaching test was performed across 8 consecutive reuse cycles to assess whether the observed activity decay was attributable to enzyme dissociation (leaching) from the support rather than intrinsic deactivation. The initial protein loading on the beads was 0.36 mg·g⁻¹ (cycle 0). After 8 cycles, the wash/elution supernatant was collected and the protein concentration was quantified using a NanoDrop spectrophotometer. The cumulative leached protein (mg·g⁻¹) was calculated by summing the amount of protein detected in the supernatants over cycles; the remaining bound protein (mg·g⁻¹) was calculated as (initial loading − cumulative leached), and the remaining bound (%) was normalized to cycle 0.

| Parameters |  |
| --- | --- |
| Protein capacity of immobilized carrier (mg/g) | 0.36 |
| Enzyme activity recovery rate of immobilized carrier (%) | 78.30 |
| Specific activity of immobilized carrier (U/g) | 18.72 |

**Table S3. Immobilized multi-enzyme cascade system parameters.**

| Sample No | 1 | 2 |
| --- | --- | --- |
| Mass of tobacco powder (g) | 0.31 | 0.30 |
| Nicotine content of tobacco extraction (mg/ml) | 1.03 | 1.01 |
| Nicotine content of tobacco leaves (%) | 3.32 | 3.35 |
| Average nicotine content of tobacco leaves (%) | 3.38 | |

**Table S4. The nicotine content in the crude extract of tobacco leaves.**
